# Diabetic eye screening in people with diabetes undergoing haemodialysis: A systematic review and meta‐analysis

**DOI:** 10.1111/dme.70157

**Published:** 2025-10-07

**Authors:** Hellena Hailu Habte‐Asres, Frehiwot Hailu, Angus Forbes, David C. Wheeler, Tunde Peto

**Affiliations:** ^1^ Florence Nightingale Faculty of Nursing, Midwifery and Palliative Care King's College London London UK; ^2^ Royal Free London NHS Foundation Trust London UK; ^3^ Colorado Technical University Colorado Springs Colorado USA; ^4^ UCL Department of Nephrology University College London, Royal Free Campus London UK; ^5^ Centre for Public Health Queen's University Belfast Belfast UK

**Keywords:** diabetic maculopathy (DM), diabetic retinopathy (DR), ESKD, haemodialysis (HD), meta‐analysis, ocular complication, systematic review

## Abstract

**Aim:**

People with diabetes receiving haemodialysis are at increased risk of diabetic retinopathy (DR) and other ocular complications. Despite this heightened vulnerability, evidence on the prevalence of such conditions in this population is limited.  This review aimed to synthesise the existing literature on the prevalence of DR and other ocular conditions, and to evaluate screening rates in people with diabetes undergoing haemodialysis.

**Methods:**

A systematic search of MEDLINE, EMBASE and CINAHL databases was conducted to identify studies reporting on the prevalence of DR, diabetic maculopathy and other ocular conditions in people with diabetes on haemodialysis. Eight articles meeting inclusion criteria were included. Pooled prevalence estimates and 95% confidence intervals (CIs) were calculated using random‐effects models.

**Results:**

The pooled prevalence of DR was 49.1% (95% CI: 23.1–75.4), while diabetic maculopathy prevalence was 32.5% (95% CI: 10.9–59.1). Other ocular complications reported included cataracts (53.4%), conjunctival calcification (63%) and corneal calcification (45.3%). Notably, 54.3% (95% CI: 35.4–72.6) of individuals on haemodialysis had not undergone eye screening in over two years.

**Conclusion:**

This review reveals a high prevalence of DR and other ocular complications in people with diabetes undergoing haemodialysis, alongside significant under‐screening. Future research should focus on developing and accessing targeted interventions to improve screening uptake in this vulnerable population.

**What's new?**

- **Screening gap**: Over half of people with diabetes on haemodialysis had not had eye screening >2 years.
- **High burden**: Nearly half have diabetic retinopathy; one in three have diabetic maculopathy.
- **Coexisting complications**: Cataracts, conjunctival and corneal calcification are common.

## 1 INTRODUCTION

Diabetic retinopathy (DR) is one of the leading causes of preventable blindness globally, particularly among the working‐age population.^1^, ^2^, ^3^ People with diabetes mellitus on haemodialysis are particularly at risk for developing DR.^4^ Both the retina and kidneys are supplied by small, low‐resistance blood vessels, which are prone to damage from fluctuations in blood glucose levels and hypertension.^5^ This shared microvascular vulnerability often leads to the concurrent progression of DR and diabetic nephropathy.^6^ These individuals face compounded risks due to poorly controlled blood glucose, hypertension and long‐term kidney dysfunction. The prevalence of DR is high among people with diabetes undergoing haemodialysis.^7^ The unique pathophysiology of diabetes in people with end‐stage kidney disease, including altered vascular changes and the effects of dialysis, can result in faster progression and more severe forms of DR, such as proliferative DR and diabetic maculopathy.^8^, ^9^

Early detection through regular eye screenings is crucial. However, attendance at DR screening is low in people with diabetes undergoing haemodialysis, resulting in undiagnosed and advanced stages of eye disease that can cause severe visual impairment or blindness.^10^ There are multiple barriers making it difficult for people with diabetes on haemodialysis to attend DR screening, such as having reduced mobility or the burden of frequent dialysis appointments impacting their availability to attend additional screening appointments.^7^, ^11^ Consequently, many opportunities for early detection and timely intervention are missed.^11^ Despite the recognised need for eye screening in haemodialysis populations, there have been few attempts to comprehensively synthesise the data on screening rates and the prevalence of ocular complications. Therefore, the aim of this study is to evaluate screening rates, the prevalence of diabetic retinopathy and other ocular complications among people with diabetes undergoing haemodialysis.

## 2 METHODS

This systematic review and meta‐analysis aimed to address this knowledge deficit. Specifically, this review sought to answer the following questions:

1. What is the prevalence of DR and diabetic maculopathy among people with diabetes undergoing haemodialysis?
2. What are the other most common ocular complications observed in individuals with diabetes on haemodialysis?
3. What are the rates of adherence to DR screening in this population?

A systematic review was conducted and reported according to the Preferred Reporting Items for Systematic reviews and Meta‐Analyses (PRISMA) and the Meta‐Analyses and Systematic Reviews of Observational Studies (MOOSE) guidelines. The protocol was registered with the International Prospective Register of Systematic Reviews (PROSPERO ID: 42025630956).

### 2.1 Data Sources

The systematic search strategy was developed with a senior health science librarian, incorporating key terms for ‘diabetic retinopathy’, ‘screening’ and ‘haemodialysis’. We conducted a time‐unrestricted search to capture all potentially relevant research, including research articles. A comprehensive search of electronic databases was initially conducted in February 2025 and updated in August 2025. The databases searched included MEDLINE, EMBASE and CINAHL. The search was limited to English‐language literature involving human participants. The search results were compiled using Covidence software, and duplicates were identified and manually reviewed for exclusion. Two reviewers (HHA and FH) independently screened the titles and abstracts of all studies to identify those that required a full‐text review. The full texts were then assessed independently for eligibility. Any disagreements were resolved through discussion, with a third reviewer available for arbitration if needed, although this was not required. Additionally, we reviewed the citations and reference lists of the included articles to identify further relevant studies or reports.

### 2.2 Study selection

#### 2.2.1 Eligibility criteria

Studies were included in the review if they specifically assessed:

1. Adults with diabetes on haemodialysis undergoing diabetic retinopathy screening, including methods such as fundus photography or handheld retinal imaging.
2. Detection rates of diabetic retinopathy, diabetic maculopathy, other ocular complications and adherence to DR screening in the haemodialysis population.
3. Randomised controlled trials (RCTs), cohort studies, cross‐sectional studies, case–control studies, evaluating and assessing diabetic retinopathy screening in haemodialysis patients.
4. Studies published in English.

Exclusion criteria were:

1. Studies that did not specifically assess DR screening in haemodialysis patients.
2. Conference proceedings, editorial opinion pieces and other non‐empirical studies.

### 2.3 Outcome

The outcomes of interest include the detection rates of diabetic retinopathy and maculopathy in people with diabetes on haemodialysis, particularly previously undiagnosed cases, as well as screening uptake and adherence rates. Secondary outcomes include the identification of other eye conditions, such as cataracts and hypertensive retinopathy.

### 2.4 Data extraction

Two authors (HHA and FH) conducted data extraction using a template designed for the study, with all data subsequently verified by other authors (AF, DW and TP). The extracted information included study characteristics (author, publication year, country and sample size), study objectives, participant demographics (age and gender), methods used to assess ocular complications and reported ocular outcomes.

### 2.5 Quality of studies

The two reviewers independently conducted a critical appraisal of the relevant studies to assess their quality using the validated Quality Assessment Tool for Observational Cohort and Cross‐Sectional Studies Scale (NHLBI), which evaluates the risk of bias in observational studies.^12^ The NHLBI Quality Assessment Tool evaluates the methodological quality of observational cohort and cross‐sectional studies. It includes 14 criteria covering aspects like research question clarity, population selection, sample size justification and exposure/outcome measurement. A summary of the areas considered in the assessment of each domain is included in the Table S1.

### 2.6 Data synthesis

The two reviewers independently extracted data on study design, publication year, follow‐up period, participant characteristics, methods, eye examination procedures, DR grading, DR grader and outcomes. The findings from the included studies were compared and presented in a results table, which informed a descriptive synthesis of the evidence retrieved.

### 2.7 Statistical Analyses

An initial descriptive analysis of the studies was conducted. Heterogeneity among the estimates was assessed using the *I* ^2^ statistic, which quantifies the proportion of variation across studies that is not due to sampling error. To address this variability as it assumes that the true effect size varies between studies rather than remaining constant.^13^ The analysis was performed using Stata software (version 17), and a pooled prevalence estimate for diabetic retinopathy, diabetic maculopathy and other ocular complications, along with a 95% confidence interval (CI), was calculated. Additionally, we estimated the level of under‐screening for DR in the haemodialysis population using a random‐effects model. Heterogeneity was assessed using the Cochrane Q statistic and quantified with the *I* ^2^ statistic, where *I* ^2^ ≥ 50% and *p* < 0.1 were considered indicative of substantial heterogeneity.^14^ Where significant heterogeneity is identified and if ten or more studies are included, we will perform subgroup analyses and meta‐regression to explore potential sources of variability.

## 3 RESULTS

**FIGURE 1:**
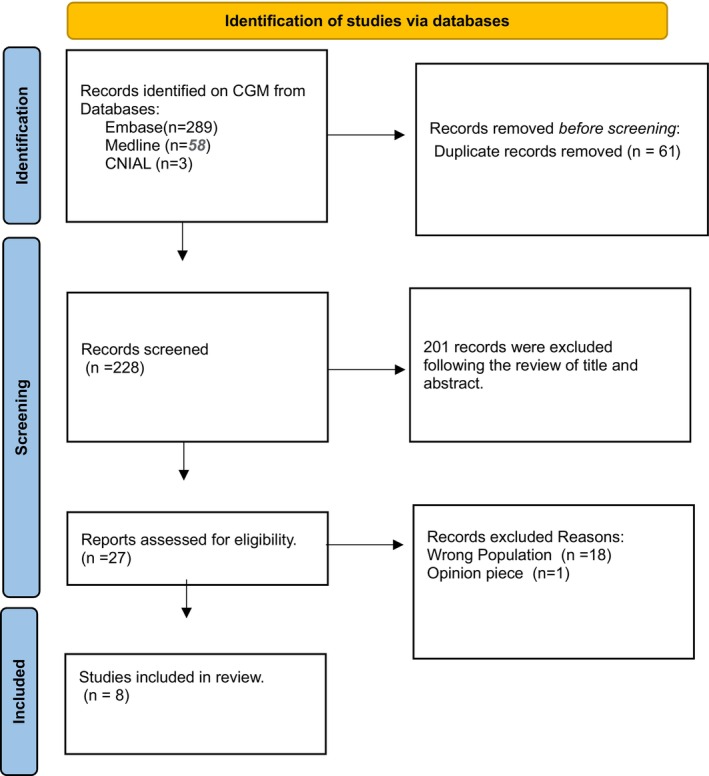
Flow diagram of study selection.

A total of 364 articles were retrieved from the databases (Embase: *n* = 289, Medline: *n* = 58 and CINAHL: *n* = 3), with 61 duplicates excluded. After screening titles and abstracts, 27 articles were shortlisted for full‐text review. Of these, eight articles were deemed eligible for inclusion. The study screening and selection process is illustrated in the PRISMA diagram (Figure 1). The quality assessment, conducted using standardised criteria, demonstrated methodological consistency across eight observational studies. Each study clearly stated its objectives, defined its population and applied valid and reliable measures for both exposures and outcomes, with exposures assessed prior to outcomes. However, none of the studies provided a sample size justification, and blinding of outcome assessors was generally absent. Repeated measurement of exposures was uncommon, and approaches to adjusting for confounding variables varied. Despite these limitations, seven studies were rated as ‘Good’ and one as ‘Fair’, suggesting overall methodological rigour, though the identified shortcomings should be considered when interpreting the findings (Table S1).

The studies were conducted across diverse regions, including the UK,^15^ Turkey,^16^ the USA,^17^ Canada,^18^ Iran,^19^ Japan,^20^ Qatar^21^ and Australia,^22^ with sample sizes ranging from 38–4173 participants, including 2983 male participants. Five of the studies had a cross‐sectional design, one utilised a survey design and another employed a cohort study design. The characteristics of the included studies are shown in Table 1. There was some variation in the study aims: Some focused on DR screening attendance, others assessed DR severity or examined other ocular complications. Most DR screening programmes are annual, with some transitioning to biennial for lower‐risk individuals. We used a two‐year cutoff to assess under‐screening in the haemodialysis population.^23^, ^24^

**TABLE 1:** Characteristics of the seven included studies.

Author & Country	Study design/period of follow‐up	Participants characteristics	Diabetes eye interventions	Outcomes
Clemens et al. (2021) (Canada)	Design: Retrospective cohort study Follow‐up: 24 months	Sample: 4173 Age: 67 ± 13 Sex: 2546 Male	Eye examinations: N/A DR grading criteria: Not reported DR grader: N/A	Primary: Identification of measurable and addressable gaps in diabetes care, specifically the lack of at least one recorded diabetic eye examination.
Cushey et al. (2022) (UK)	Design: Cross‐sectional Audit Follow‐up: N/A	Sample: 132 Age: 33–91 years Sex: 88 Male	Eye examinations: Fundus images centred on the macula and optic disc were captured for each eye using a Canon CR‐2 non‐mydriatic tabletop camera and an Optomed Aurora handheld fundus camera DR grading criteria: Diabetic retinopathy was graded according to the United Kingdom national grading definitions: no visible retinopathy (R0), background retinopathy (R1), preproliferative retinopathy (R2), active proliferative retinopathy (R3A), stable proliferative retinopathy (R3S), no maculopathy requiring referral (M0) and maculopathy requiring further evaluation (M1). DR grader: Two graders and subset were graded by AI	Primary: attendance at the Diabetic Eye Screening Programme and the severity of diabetic retinopathy (DR) among patients with diabetes attending haemodialysis units
Egeolu et al. (2023) (USA)	Design: Cross‐sectional study Follow‐up: N/A	Sample: 93 Age: Not reported Sex: 47 Male	Eye examinations: OPTOMED handheld camera DR grading criteria: No apparent, mild non‐proliferative, moderate non‐proliferative, severe non‐proliferative and proliferative DR DR grader: Retinal specialist and artificial intelligence software	Primary: To determine the prevalence of DR
El‐Menyar et al. (2012) (Qatar)	Design: Cross‐sectional study Follow‐up: 3 years	Sample: 252 Age: 57 ± 15 Sex: 101 Male	Eye examinations: Diabetic retinopathy (DR) was assessed using slit‐lamp biomicroscopy with a + 90 D lens and/or indirect ophthalmoscopy DR grading criteria: DR was defined as the presence of typical microvascular signs DR grader: Not reported	Primary: All‐cause morbidity and mortality over a 3‐year period.
Estevez et al. (2024) (Australia)	Design: Cross‐sectional study Follow‐up: N/A	Sample: 215 Age: 65.1 ± 13.5 Sex: 108 Male	Eye examinations: Distance visual acuity (VA), Intraocular pressure was measured using a rebound tonometer. The anterior segment was photographed or examined using direct ophthalmoscopy, with a slit beam for the cornea and a round beam for lens status. A single 45‐degree, macula‐centred colour retinal photograph was taken of each eye using the Topcon 3D‐1 OCT Maestro (Topcon, Japan) for the Southern Adelaide region and the Canon CR32 (Canon Medical Systems, USA) for the Central Australia region to assess the posterior eye. DR grading criteria: DR was graded based on the simplified Wisconsin scale as; No DR, minimal, mild, moderate or severe non‐proliferative DR or PDR DR grader: By trained optometrists, research assistants, ophthalmology trainees or supervising consultants using standardised protocols	Primary: To determine the prevalence of vision impairment and blindness.
Kianersi et al. (2019) (Iran)	Design: Cross‐sectional study Follow‐up: N/A	Sample: 121 Age: 51.59 ± 16.01 Sex: 68 Male	Eye examinations: Eye examinations included assessing best‐corrected visual acuity (BCVA) and performing direct fundus biomicroscopy to evaluate the retina, optic disc, macula and vitreous. Intraocular pressure (IOP) was measured using a slit‐lamp‐mounted Goldmann applanation tonometer. Biomicroscopic examination was conducted with a slit‐lamp, and fundus evaluation was done using 90 diopter . DR grading criteria: Not reported DR grader: Vitreo‐retina specialist	Primary: The prevalence of ocular abnormalities, including cataract, conjunctival calcification, corneal calcification and optic atrophy.
Kimura et al. (2022) (Japan)	Design: Survey Follow‐up: N/A	Sample: 38 Age: 68.7 ± 12.0 Sex: 25 Male	Eye examinations: N/A DR grading criteria: Not reported DR grader: N/A	Primary: the rate of ophthalmology consultations among people with diabetes undergoing haemodialysis
Sariyeva et al. (2020) (Turkey)	Design: Cross‐sectional study Follow‐up: N/A	Sample: 71 Age: 60.7 ± 13.0 Sex: 39 Male	Eye examinations: slit‐lamp evaluation and fundoscopy. DR grading criteria: Not reported DR Grader: Not reported	Primary: The frequency of ocular manifestations, including diabetic retinopathy, cataract, corneal and conjunctival deposits, hypertensive retinopathy, optic atrophy and glaucoma.

The studies frequently utilised retinal imaging and ocular health assessments, particularly for DR, employing various equipment, including the Canon CR‐2, Topcon 3D‐1 OCT Maestro and Optomed Aurora handheld cameras. For DR grading, different systems were applied, including the UK National Grading System,^15^ a simplified scale^17^ and the WHO protocol.^22^ Two studies^15^, ^17^ incorporated artificial intelligence (AI) to support grading, with only one study reporting varying levels of agreement between human graders and AI.

### 3.1 Meta‐analysis results

The main outcomes for each of the studies can be found in Table 2.

**TABLE 2:** Reported outcomes from the included studies.

Study	Aim	Method of screening	DR Screening attendance	Prevalence of ocular conditions
Clemens et al. (2021) (Canada)	To identify diabetes care gaps and modifiable predictors among patients receiving in‐centre haemodialysis.	Medical Chart Review: Dialysis Database	2201 (53%) No evidence of retinopathy screening	Diabetic retinopathy: DR: Retinopathy: 473 (11)
Cushey et al. (2022) (UK)	To assess attendance at the Diabetic Eye Screening Program, diabetic retinopathy severity and the use of handheld retinal imaging in people with diabetes undergoing haemodialysis	Canon CR‐2 non‐mydriatic tabletop camera and Optomed Aurora handheld camera	42 had an interval of >2 years 21 had an interval of 3 years 26 had >4 years of nonattendance 21 never attended the DR screening 3 Too sick to attend	Diabetic retinopathy: No DR (R0): 28 (21%) Background DR (R1): 36 (27%) STDR (R2, R3A, R3S): 30 (23%) Diabetic maculopathy: No maculopathy (M0): 77 (58%) Maculopathy (M1): 14 (11%) Progression DR: No DR to background DR: 7 Background to STDR: 9 STDR to no perception of light: 1
Egeolu et al. (2023) (USA)	To determine the prevalence of diabetic retinopathy (DR) among African Americans with diabetes and undergoing dialysis.	DR was based on a review of medical records and/or a positive photograph with a portable handheld device (Optomed Aurora handheld camera)	61% within 1 year 20% within 2 years 14% more than 2 years 20 had no previous report in electronic medical records	Diabetic retinopathy: Mild DR: 33% Moderate: 9.6% Severe DR: 57.4% Visual impairment Normal visual acuity: 43% Moderate visual impairment: 45% Severe visual impairment: 12%
El‐Menyar et al. (2012) (Qatar)	To assess prevalence and outcomes of DR in HD patients.	slit‐lamp biomicroscopy with a + 90 D lens and/or indirect ophthalmoscopy	Not reported	Diabetic retinopathy: DR (any grade): 113 (45%)
Estevez et al. (2024) (Australia)	To examine the prevalence of vision impairment and diabetic retinopathy in Indigenous and non‐Indigenous Australians with type 2 diabetes undergoing haemodialysis.	Self reported information was cross‐checked with medical records at the haemodialysis clinic. Slit beam for the cornea and a round beam for lens status. Canon CR32	Not reported	Diabetic retinopathy: No retinopathy: 14.9 (10.7–20.3) Minimal NPDR: 7.9 (5.0–12.4) Mild NPDR: 18.7 (13.9–24.4) Moderate NPDR: 21.9 (16.9–27.9) Severe NPDR: 9.3 (6.1–14.0) PDR: 27.0 (21.4–33.3) Diabetic maculopathy: Any maculopathy: 49.2 (42.6–56.0) Vision impairment Unilateral VI 50 (23.5, 18.1–30.7) Bilateral VI 25 (11.7, 8.8–17.8) Unilateral blindness: 31 (14.2, 10.1–20.5) Bilateral blindness: 8 (3.7, 2.1–8.0)
Kianersi et al. (2019) (Iran)	To investigate the frequency of ocular manifestations in haemodialysis (HD) patients	Biomicroscopy was performed with a slit‐lamp, and direct fundus examination used 90D lenses in a seated position.	Not reported	Ocular complication: Conjunctival calcification: 78 (32.2) Corneal calcification: 78 (32.2) Glaucoma: 18 (7.4) Cataract: 71 (58.7) Optic atrophy: 46 (19) Retinal vein occlusion: 6 (2.5) Proliferative diabetic retinopathy:70 (28.9) Macular oedema: 51 (21.1) Vitreous haemorrhage: 28 (11.6) Hypertensive retinopathy 54 (22.3)
Kimura et al. (2022) (Japan)	To investigate the status of ophthalmology consultations and the use of the diabetic eye notebook (DEN) among haemodialysis patients with diabetes	Participants were asked whether they had ever seen an ophthalmologist.	22 (58%) patients had regular ophthalmology consultations	Ocular complication: Diabetic retinopathy: 19 Cataract: 17 Others: 5
Sariyeva et al. (2020) (Turkey)	To investigate the frequency of ocular manifestations in haemodialysis (HD) patients	Slit‐lamp examination and fundoscopy. Patients were screened for corneal and conjunctival deposits, diabetic retinopathy, hypertensive retinopathy, cataracts, optic atrophy and glaucoma.	Not reported	Ocular complication: Conjunctival calcification: 43 (60.6%) Corneal calcification: 11 (15.5%) Glaucoma: 5 (7%) Cataract: 36 (50.7%) Proliferative diabetic retinopathy: 15 (21.1%) Optic atrophy: 11 (15.5%) Hypertensive retinopathy: 10 (14.1%)/

#### 3.1.1 Suboptimal diabetic retinopathy screening attendance

**FIGURE 2:**
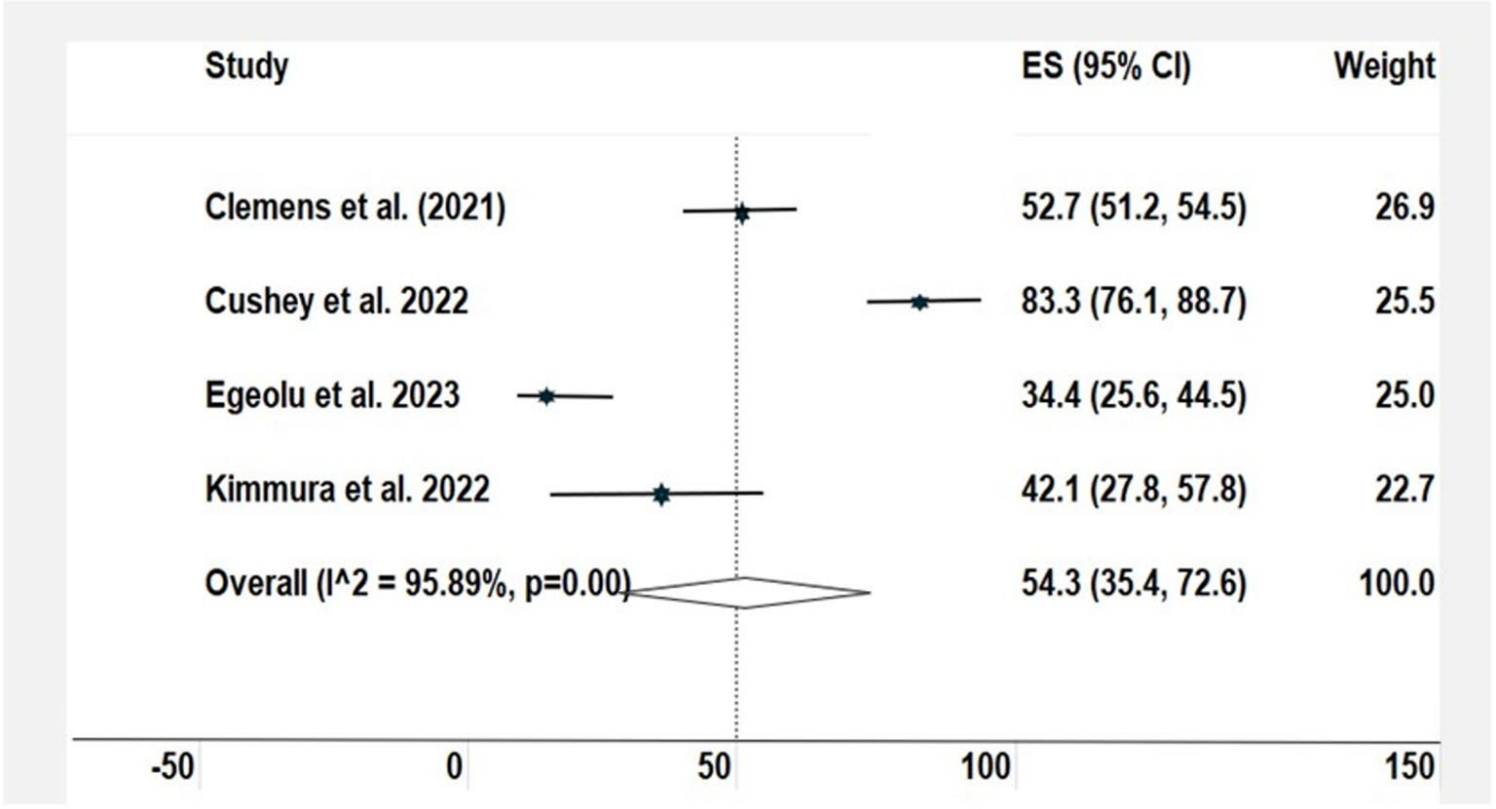
Estimated prevalence of suboptimal retinopathy screening (no screening ≥2) years) in patients on haemodialysis.

The meta‐analysis pooled data from four studies^15^, ^17^, ^18^, ^25^ were used to estimate the prevalence of individuals with diabetes on haemodialysis who had not been screened for more than 2 years. The pooled estimate was 54.3% (95% CI: 35.4–72.6). Significant heterogeneity was observed (*I* ^2^ = 95.9%, χ^2^ = 73.07, *p* < 0.001), indicating variability in screening rates across studies (Figure 2).

#### 3.1.2 Prevalence of diabetic retinopathy

**FIGURE 3:**
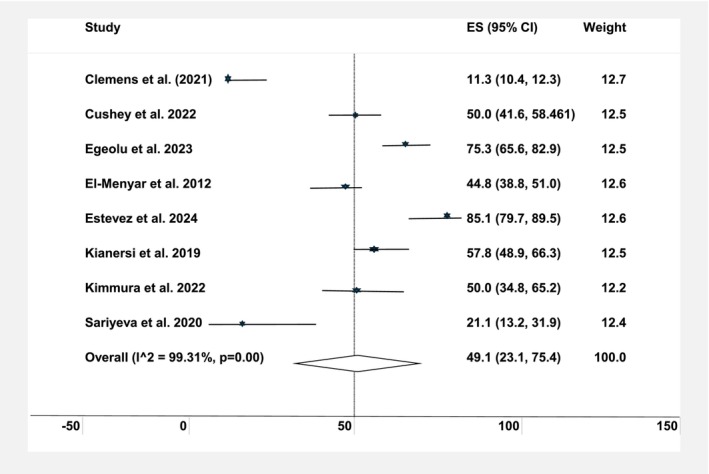
Estimated prevalence of diabetic retinopathy in patients on haemodialysis.

Data from seven studies^15^, ^16^, ^17^, ^18^, ^19^, ^20^, ^21^, ^22^ were pooled to estimate the prevalence of diabetic retinopathy in haemodialysis patients, with a pooled estimate of 49.1% (95% CI: 23.1–75.4). Significant heterogeneity was observed (*I* ^2^ = 99.4%, *p* < 0.001), indicating considerable variation in DR prevalence rates across the studies (Figure 3).

#### 3.1.3 Prevalence of diabetic maculopathy

The meta‐analysis combined data from three studies,^15^, ^19^, ^22^ yielding a pooled prevalence of 32.5% (95% CI: 10.9–59.1). The individual prevalence rates reported were 10.6% (95% CI: 6.4–17.0) in Cushey et al.,^15^ 49.3% (95% CI: 42.7–55.9) in Estevez et al.^22^ and 42.2% (95% CI: 33.7–51.1) in Kianersi et al.^19^ However, the meta‐analysis revealed substantial heterogeneity (*I* ^2^ = 97.0%, *p* < 0.001), reflecting significant variability across studies in the reported prevalence rates.

#### 3.1.4 Ocular complications in the haemodialysis population

The meta‐analysis pooled data from multiple studies to estimate the prevalence of various ocular complications in haemodialysis patients:

- Cataracts: Data from three studies^16^, ^19^, ^20^ were pooled, yielding a prevalence rate of 53.4% (95% CI: 45.6–61.0; *p* < 0.001), indicating a high prevalence of cataracts in this population. Heterogeneity was low (*I* ^2^ = 24.49%, *p* = 0.27).
- Conjunctival Calcification: Data from two studies^16^, ^19^ were pooled, yielding a prevalence of 63.0% (95% CI: 56.1–69.8; *p* < 0.001), indicating a high prevalence of conjunctival calcification.
- Corneal Calcification: Data from two studies^16^, ^19^ were pooled, showing a prevalence of 45.3% (95% CI: 38.2–52.4; *p* < 0.001), indicating a notable occurrence of corneal calcification.
- Optic Atrophy: Data from two studies^16^, ^19^ were pooled, resulting in a prevalence of 29.0% (95% CI: 22.74–35.7; *p* < 0.001), highlighting a significant prevalence of optic atrophy in this population.
- Hypertensive Retinopathy: Data from two studies^16^, ^19^ were pooled, yielding a prevalence of 32.2% (95% CI: 25.8–39.1; *p* < 0.001), highlighting a significant prevalence of hypertensive retinopathy among haemodialysis patients.

## 4 DISCUSSION

This systematic review and meta‐analysis provide valuable insights into the detection and prevalence of DR, diabetic maculopathy and other ocular complications in people with diabetes undergoing haemodialysis. This is the first known review to estimate the prevalence of these conditions in this high‐risk group, which is often underserved in screening programmes. Our findings are clinically relevant in light of guidance from the National Institute for Health and Care Excellence (NICE),^26^, ^27^ American *Diabetes Association* (ADA) Standards of Medical Care in Diabetes^28^ and the WHO's *Diabetic Retinopathy Screening: A Short Guide*,^29^ all of which include diabetic retinopathy screening as a core quality indicator and emphasise risk‐based, patient‐centred care. However, current care pathways may not adequately accommodate the complex needs and logistical constraints faced by people with diabetes undergoing haemodialysis.

This review highlights a significant gap in DR screening among people with diabetes undergoing haemodialysis. Despite the proven benefits of early detection in reducing the risk of vision loss,^7^ 54.3% of this population have either never been screened or have not been screened within the past two years. This level of under‐screening is concerning and reflects missed opportunities for early intervention, as well as broader inequalities in care provision. Current clinical guidelines, including those from the American Diabetes Association and the UK's national screening programme, recommend annual to biennial DR screening depending on individual risk—supporting the use of a two‐year threshold to define suboptimal screening. While screening rates in the general population in high‐income countries typically range from 65% to 80%, uptake is markedly lower among those on haemodialysis. Available estimates suggest that fewer than 50% of patients in Canada, less than 60% in Japan and only around 40% in North Central London receive regular eye examinations.^18^, ^20^, ^30^ These disparities underline the urgent need to develop more accessible, integrated screening pathways that address the complex healthcare needs of this vulnerable group.

The pooled prevalence of DR and diabetic maculopathy among people with diabetes undergoing haemodialysis was notably high, at 49.1% and 32.5%, respectively, reflecting the considerable microvascular burden in this population. This is not unexpected, given the shared pathophysiology of microvascular damage, to which individuals receiving haemodialysis are particularly vulnerable. However, substantial heterogeneity was observed across studies (*I* ^2^ = 99.4%, *p* < 0.001), indicating wide variation in reported prevalence rates. This variability is likely influenced by differences in patient demographics, DR grading protocols and screening technologies. Although a random‐effects model was used to account for this, the findings should be interpreted with caution. The small number of included studies limited our ability to conduct subgroup analyses or meta‐regression, restricting further exploration of these sources of heterogeneity. As such, the results should be considered hypothesis‐generating. Future research should prioritise the use of standardised DR grading systems, harmonised screening methodologies and integration of DR screening metrics into diabetes care quality frameworks to support more consistent reporting, improve comparability across studies and enable robust clinical benchmarking.

Beyond DR, people with diabetes on haemodialysis experience a substantial burden of additional ocular conditions. This review found high prevalence rates of cataracts (53.4%), conjunctival calcification (63.0%) and corneal calcification (45.3%), highlighting the wider impact of microvascular and metabolic disturbances in this group. These complications may be driven by chronic hyperglycaemia, disrupted calcium‐phosphate metabolism, medication effects and the physiological demands of dialysis. For instance, cataracts are frequently observed in diabetes and chronic kidney disease due to long‐term metabolic stress and altered ocular perfusion, while ocular surface calcifications may reflect underlying mineral imbalances linked to renal dysfunction.^31^

These findings emphasise the need for holistic ocular assessment, extending beyond DR screening alone. Given the complexity of care and frequent clinical appointments, offering eye health services during dialysis sessions using portable or AI‐enabled technologies could improve access and reduce patient burden. Embedding such approaches into routine care would support coordinated service delivery, in line with the NHS Long Term Plan^32^ and the American Diabetes Association's emphasis on integrated models and may facilitate better engagement with national eye screening programmes.

This review highlights both the diversity of DR screening methods and a critical evidence gap in diabetes care for individuals undergoing haemodialysis. Screening approaches varied across studies, including fundus photography, slit‐lamp examinations and handheld retinal imaging devices.^15^, ^17^, ^22^ While fundus photography is considered the gold standard, handheld devices represent a promising alternative, particularly in dialysis settings where traditional off‐site appointments may be challenging. Their portability and ease of use offer a practical solution for increasing screening uptake within dialysis units.

The findings have important implications for clinical commissioning, service design and integrated models of care. Embedding DR screening into routine dialysis pathways supported by accessible technologies and multidisciplinary collaboration may reduce health inequalities and prevent avoidable vision loss. Aligning these efforts with international standards and the priorities outlined in the NICE and American Diabetes Association guidelines would support broader health system goals, including the reduction of diabetes‐related complications and the promotion of equitable, value‐based care.

## 5 STRENGTH AND LIMITATIONS

This review is strengthened by its adherence to PRISMA and MOOSE guidelines and the use of validated appraisal tools, ensuring methodological rigor. Nonetheless, several limitations must be considered. The inclusion of only eight studies constrained opportunities to conduct planned subgroup analyses or investigate sources of variability through meta‐regression. High heterogeneity, as reflected by the *I* ^2^ values, limits the applicability of pooled estimates and likely stems from differences in geography, study design, sample characteristics and screening protocols. Furthermore, inconsistencies in grading approaches across studies point to the need for standardised methodologies in future research. Despite these constraints, the review advances understanding of the ocular disease burden in individuals with diabetes receiving haemodialysis and identifies priorities for improving evidence and care pathways in this underserved group.

## 6 IMPLICATIONS FOR FUTURE RESEARCH AND CLINICAL PRACTICE

The findings highlight the urgent need for integrated, patient‐centred DR screening models within dialysis settings. Future research should focus on evaluating the effectiveness of in‐unit screening using portable retinal cameras or AI‐assisted grading systems, which could reduce logistical barriers and enhance patient participation. Further studies should also compare the effectiveness of various screening methods, such as handheld devices and AI‐driven grading, to identify the most accurate and efficient approaches for this high‐risk group. Additionally, exploring the impact of early DR detection on patient outcomes, particularly visual acuity and quality of life, is crucial. Understanding the long‐term benefits of timely intervention will be pivotal in advocating for routine screening in haemodialysis units and in developing more inclusive, equitable frameworks for diabetes care.

## 7 CONCLUSION

This systematic review and meta‐analysis provide robust evidence of the significant burden of ocular complications, particularly DR and maculopathy, in people with diabetes undergoing haemodialysis. Addressing barriers to screening through integrated, technology‐enabled models, such as incorporating handheld retinal imaging and exploring the use of artificial intelligence, could significantly improve access to care and patient outcomes. Aligning these models with NICE and ADA diabetes care standards may facilitate their implementation and help close critical care gaps, reducing disparities and protecting vision in this vulnerable population.

## AUTHOR CONTRIBUTIONS

HHA conceived and designed the review; developed the protocol; conducted the literature search; led the screening, data extraction and quality assessment; performed the meta‐analysis; and drafted the manuscript. FH contributed to title and abstract screening, data extraction, and supported the synthesis and interpretation of findings. AF provided methodological guidance, supervised the review process and contributed to the critical revision of the manuscript. TP contributed expert knowledge on diabetic retinopathy and eye screening and critically revised the manuscript. DCW contributed expertise on kidney disease, supervised the clinical framing of the review, critically revised the manuscript and is the guarantor of the work. All authors reviewed and approved the final version of the manuscript and agreed to be accountable for the accuracy and integrity of the work.

## FUNDING INFORMATION

The authors received no financial support for the research, authorship and/or publication of this article.

## CONFLICT OF INTEREST STATEMENT

HH‐A received speaker honoraria from AstraZeneca and Bayer. FH AF and TP have declared no conflicts of interest. DW has an ongoing consultancy contract with AstraZeneca and has received payments for consultancy and/or speaking activities from multiple companies, including Amgen, Astellas, Bayer, Boehringer Ingelheim, Eledon, GSK, Galderma, Gilead, Janssen, Mundipharma, Menarini, MSD, NovoNordisk, Pharmacosmos, Tricida and Vifor.

## Supporting information


Data S1:
